# Changes of Enterocyte Morphology and Enterocyte: Goblet Cell Ratios in Dogs with Protein-Losing and Non-Protein-Losing Chronic Enteropathies

**DOI:** 10.3390/vetsci10070417

**Published:** 2023-06-27

**Authors:** David Díaz-Regañón, Vojtech Gabriel, Vanessa Livania, Dongjie Liu, Basant H. Ahmed, Addison Lincoln, Hannah Wickham, Abigail Ralston, Maria M. Merodio, Dipak K. Sahoo, Christopher Zdyrski, David K. Meyerholz, Jonathan P. Mochel, Karin Allenspach

**Affiliations:** 1 Department of Animal Medicine and Surgery, Faculty of Veterinary Medicine, Complutense University of Madrid, 28040 Madrid, Spain; 2 SMART Lab, Department of Biomedical Sciences, Iowa State University, Ames, IA 50011, USA; vojt.gabriel@gmail.com (V.G.); vanessa-livania@uiowa.edu (V.L.); djliu@iastate.edu (D.L.); atbasant@iastate.edu (B.H.A.); agrace@iastate.edu (A.L.); hannah.marie.wickham@gmail.com (H.W.); mmaria@iastate.edu (M.M.M.); czdyrski@iastate.edu (C.Z.); jmochel@iastate.edu (J.P.M.); 3 3D Health Solutions Inc., Ames, IA 50010, USA; abby.ralston@3dhealth.solutions; 4 Department of Veterinary Clinical Sciences, Iowa State University, Ames, IA 50011, USA; dsahoo@iastate.edu; 5 Department of Pathology, Carver College of Medicine, University of Iowa, Iowa City, IA 52242, USA; david-meyerholz@uiowa.edu

**Keywords:** chronic enteropathies, inflammatory bowel disease, dog, protein-losing enteropathies, goblet cells, enterocytes, intestines, morphometry

## Abstract

**Simple Summary:**

Recent studies have emphasized the importance of intestinal mucosal architectural changes in chronic enteropathies such as celiac disease and chronic environmental enteropathies in human beings. This current study sought to examine changes in the morphology of the intestinal enterocytes and the proportion of mucus-producing cells (goblet cells) to enterocytes in the small and large intestines of dogs with chronic enteropathies (CE). Tissue samples from healthy dogs and dogs with CE with and without protein-losing enteropathy (PLE), were assessed. Healthy adult dogs presented with progressively shorter enterocytes from duodenum to jejunum to ileum, while juvenile dogs presented with increasing enterocyte height in the same direction. Dogs with CE had taller cells in the duodenum, while those with PLE had decreased duodenal enterocyte height compared to healthy dogs. The width of the intestinal cells was also reduced in CE dogs compared to healthy dogs. Additionally, the ratio of goblet cells to intestinal cells in CE was decreased in the colon when compared to healthy dogs. This study demonstrates that dogs with chronic enteropathies, similar to celiac disease in people, present with significant alterations in the size of the enterocytes and a reduced proportion of mucus-producing cells in the colon.

**Abstract:**

This study aimed to assess the morphometry of enterocytes as well as the goblet cell-to-enterocyte ratio in different intestinal segments of dogs with chronic enteropathies (CE). Histopathological intestinal samples from 97 dogs were included in the study (19 healthy juveniles, 21 healthy adults, 24 dogs with protein-losing enteropathy (PLE), and 33 CE dogs without PLE). Healthy adult small intestinal enterocytes showed progressively reduced epithelial cell height in the aboral direction, while juvenile dogs showed progressively increased epithelial cell height in the aboral direction. CE dogs had increased epithelial cell height in the duodenum, while PLE dogs had decreased epithelial cell heights compared to healthy adult dogs. Both the CE and PLE dogs showed decreased enterocyte width in the duodenal segment, and the ileal and colonic enterocytes of CE dogs were narrower than those of healthy adult dogs. CE dogs had a lower goblet cell-to-enterocyte ratio in the colon segment compared to healthy dogs. This study provides valuable morphometric information on enterocytes during canine chronic enteropathies, highlighting significant morphological enterocyte alterations, particularly in the small intestine, as well as a reduced goblet cell-to-enterocyte ratio in the colon of CE cases compared to healthy adult dogs.

## 1. Introduction

Canine chronic enteropathies (CE) are diagnosed based on the presence of chronic gastrointestinal (GI) signs (i.e., vomiting, diarrhea, weight loss) lasting more than three weeks, histopathologic evidence of benign intestinal mucosal inflammation, and the exclusion of other underlying causes [1,2,3]. Canine CE are currently retrospectively diagnosed based on the response to treatment. Thus, CE are subclassified as food-responsive (FRE), antibiotic-responsive (ARE), immunosuppressant-responsive (IRE), also referred to as inflammatory bowel disease (IBD), and finally, a group that are non-responsive (NRE) [3,4,5,6].

Protein-losing enteropathy (PLE) is specified as a severe form of CE in dogs when the underlying diagnosis is characterized by severe inflammation [7,8], with accompanying protein loss into the GI lumen [6]. Dogs with PLE most commonly present with chronic small intestinal diarrhea, vomiting, anorexia, weight loss, and systemic complications (e.g., ascites, peripheral edema) secondary to severe hypoalbuminemia [1,6,7]. The diagnosis of CE and PLE requires histopathological evaluation of endoscopically collected biopsies from the GI tract after an appropriate and complete diagnostic workup that allows the exclusion of other GI or extra-GI etiologies that can mimic CE [4].

Histopathologic assessment of the biopsies is based on the inflammatory infiltration of cells into the lamina propria and epithelium as well as morphological features of the epithelia themselves [2,9]. Since the World Small Animal Veterinary Association (WSAVA) International Gastrointestinal Standardization Group established the guidelines for the evaluation of GI inflammation in companion animals in 2010 [2], clinical validation of the WSAVA scoring index in dogs with CE has been the focus of multiple studies carried out in the last decade [10,11,12,13,14,15,16]. However, many of these studies have failed to show a strong association between clinical activity in dogs with CE [1,17] and scores assigned through the WSAVA index [2]. In this context, some authors have developed simplified histopathologic scoring systems to ensure a better correlation among histopathological findings and clinical activity indices [11,12]. One aspect that became clear with more groups using the WSAVA index is the importance of cellular and architectural changes of the epithelium in CE [10,11]. However, only a few studies compared histopathologic findings from dogs with CE with and without hypoalbuminemia. Two of these studies showed that dogs with PLE presented with more severe architectural lesions including villous stunting, epithelial injury, crypt distension, and lacteal dilatation, as well as increased intraepithelial lymphocyte numbers than dogs with normo-albuminemia [1,10]. Furthermore, alterations in the architecture of the epithelium including not only the villi, but also the microvilli and the height and width of the enterocytes have been found in celiac disease (CD) as well as environmental enteric dysfunction (EED) in humans [18,19]. In addition, the numbers of goblet cells in different areas of the intestine have been found to be disturbed in various chronic intestinal diseases in humans [19,20].

Our goal was therefore to deepen our knowledge base of architectural changes in the epithelium of dogs with CE, and to investigate morphological features (width and height) of the enterocytes as the functional unit of the intestinal epithelium in dogs with CE and PLE. Moreover, we set out to investigate the ratio of goblet cells to enterocyte numbers in the different intestinal segments of dogs with CE. We hypothesized that enterocytes of dogs with CE and PLE will present with significant morphological alterations, especially affecting the small intestine, and that the ratio of goblet/enterocyte numbers will be altered in CE cases in comparison to healthy dogs.

## 2. Materials and Methods

### 2.1. Dogs Inclusion

A total of 57 dogs with CE and 40 healthy dogs were included in this retrospective study. The group of dogs with CE was further divided into CE (with normal serum albumin concentrations; range: 2.7–4 g/dL) (*n* = 33), and PLE (with decreased serum albumin concentrations; <2.7 g/dL or signs of ascites or peripheral edema) (*n* = 24). The CE and PLE patients were diagnosed at the Lloyd Veterinary Medical Center (Iowa State University) under supervision of ACVIM/ECVIM board certified clinicians. CE patients showed chronic GI signs, benign mucosal inflammation, and lack of other causes leading to inflammatory changes. Furthermore, the dogs showed insufficient response to anthelmintics and diet changes alone, while responding to immunosuppressive treatment. PLE patients suffered from hypoalbuminemia as part of panhypoproteinemia, and protein-losing nephropathy and liver failure were excluded as underlying causes for hypoalbuminemia. After obtaining intestinal biopsies in each of the dogs, immunosuppressive therapy was administered if deemed necessary by the clinician.

Forty healthy dogs that underwent intestinal biopsies for unrelated reasons were included as a healthy control group and were grouped into adults (one year of age or higher; *n* = 21) or juvenile (less than a year; *n* = 19) [21].

All protocols were approved by Iowa State University Institutional Animal Care and Use Committee (References: IACUC-18-065, IACUC-19-337, and IACUC-19-102).

### 2.2. Morphometrical Study of the Enterocytes and Goblet Cell-to-Enterocyte Ratio

Endoscopic intestinal biopsies or necropsy samples from the duodenum, jejunum, ileum, and colon were obtained from the group of healthy dogs. For CE dogs, jejunal biopsies were not available, as endoscopic biopsies cannot be obtained from the jejunum.

Paraffin-embedded biopsies and tissues were sliced and stained with hematoxylin-eosin (H&E) and Alcian blue (AB) in the Veterinary Diagnostic Laboratory at Iowa State University. Ten random pictures of each slide were captured at 40× objective magnification with the Olympus CX21 microscope using cellSense v. 1.16 software (Olympus Life Sciences, Center Valley, PA, USA). A standardized measuring and counting protocol was applied using a post-examination method of masking the observer to group assignment [22]. The height and width of enterocytes and the ratio between goblet cells and enterocytes was investigated in the various intestinal segments using ImageJ software (National Institute of Health, Bethesda, MA, USA) [23]. When measuring the height of each cell, the microvilli and basal membrane of the enterocytes were identified. For width measurements, the nucleus was used as a reference point and mid-nuclear measurements were made. A total of 100 height measurements and 100 width measurements of the enterocytes were made per tissue site using the H&E slides. In addition, a minimum of 2000 cells were counted to calculate the goblet cell-to-enterocyte ratio using the slides stained with AB.

The following biopsy samples were available for the study: 19 duodenum, 14 jejunum, 19 ileum, and 16 colon samples from healthy juvenile dogs; 21 duodenum, 3 jejunum, 15 ileum, and 19 colon samples from healthy adult dogs; 28 duodenum, 24 ileum, and 24 colon samples from CE dogs, and 21 duodenum, 14 ileum, and 10 colon samples from PLE patients were included for the measurement of the enterocytes (H&E staining). In addition, 19 duodenum, 13 jejunum,19 ileum, and 18 colon samples from healthy juvenile dogs; 19 duodenum, 4 jejunum, 15 ileum, and 16 colon samples from healthy adult dogs; 28 duodenum, 16 ileum, and 24 colon samples from CE dogs, and 21 duodenum, 7 ileum, and 9 colon samples from PLE patients were included for the goblet cell-to-enterocyte ratio analysis (AB staining).

### 2.3. Laboratory and Clinical Data

Information from each CE and PLE patient included the following: sex, neuter status, breed, age, body condition scoring (BCS), body weight (BW); clinical activity indexes: CIBDAI [17] and CCECAI [1]; complete blood count (CBC) and biochemistry profile, basal cortisol and GI panel including serum folate, cobalamin (Vitamin B12), Pancreatic Lipase Immunoreactivity (PLI), and Trypsin-like Immunoreactivity (TLI) and urine protein by dipstick evaluation. All variables were statistically compared between CE and PLE groups and correlated with the CCECAI index.

### 2.4. Statistical Analysis

Statistical analyses were performed using GraphPad Prism 9.4 software. Data were cleaned of all outliers using the accepted ROUT method (Q = 10%). Cleaned data were tested for normality via D’Agostino–Pearson omnibus normality test. Parametric data were tested with a multiple unpaired *t*-test with the two-stage step-up method of Benjamini, Krieger and Yekutieli. Correlation of the adjusted data was investigated using a nonparametric Spearman test. Statistical significance was assessed using a two-way *t*-test. Statistical significance (*p* < 0.05) was analyzed using an ordinary One-Way ANOVA multiple comparison.

## 3. Results

### 3.1. Animal Charactheristics

The healthy juvenile dogs were mixed-breed dogs (except for 1 Beagle), with a median age of 1.03 months (range: newborn to 8.17 months) and included 8 females and 11 males. The healthy adult group were Beagle dogs (except for 1 mixed-breed dog), with a median age of 2.5 years (range 1–2.71 years) and included 14 females and 7 males.

The CE with PLE include dogs from Yorkshire Terrier (*n* = 4), Vizsla (*n* = 3), Basset Hound, Bernese Mountain, Bichon Frise, Cocker Spaniel, English Bulldog, English Pointer, Pitbull, Pug, Soft Coated Wheaten Terrier, Toy Poodle and Welsh Terrier (*n* = 1 each) breeds, and mixed-breed dogs (*n* = 6) with a median age of 8.20 years (range: 2.5 to 13.00) and included 11 females and 13 males.

The CE without PLE included dogs from Labrador Retriever (*n* = 4), Boston Terrier (*n* = 3), Pembroke Welsh Corgi (*n* = 2), Staffordshire Bull Terrier (*n* = 2), Boxer, Cardigan Welsh Corgi, Cavalier King Charles, Cocker Spaniel, French Bulldog, German Shepherd, German Shorthair Pointer, Golden Retriever, Great Pyrenees, Jack Russell Terrier, Norwegian Elkhound, Pitbull, Shih Tzu, Siberian Husky, Silky Terrier, Skye Terrier, Toy Poodle, Whippet, and Yorkshire Terrier (*n* = 1 each) breeds, and mixed-breed dogs (*n* = 4), with a median age of 8.8 years (range: 1.00 to 14.4 years), and included 15 females and 17 males.

No differences were found in age between CE with or without PLE (*p* = 0.99) or between healthy adult and juvenile dogs (*p* = 0.059). However, statistically significant differences were found between healthy adult and CE and PLE dogs (*p* < 0.0001 and *p* = 0.0002, respectively), and between healthy juvenile and CE and PLE dogs (*p* < 0.0001 in both).

### 3.2. Height and Width of the Enterocytes, and Goblet Cell-to-Enterocyte Ratio

**Figure 1:**
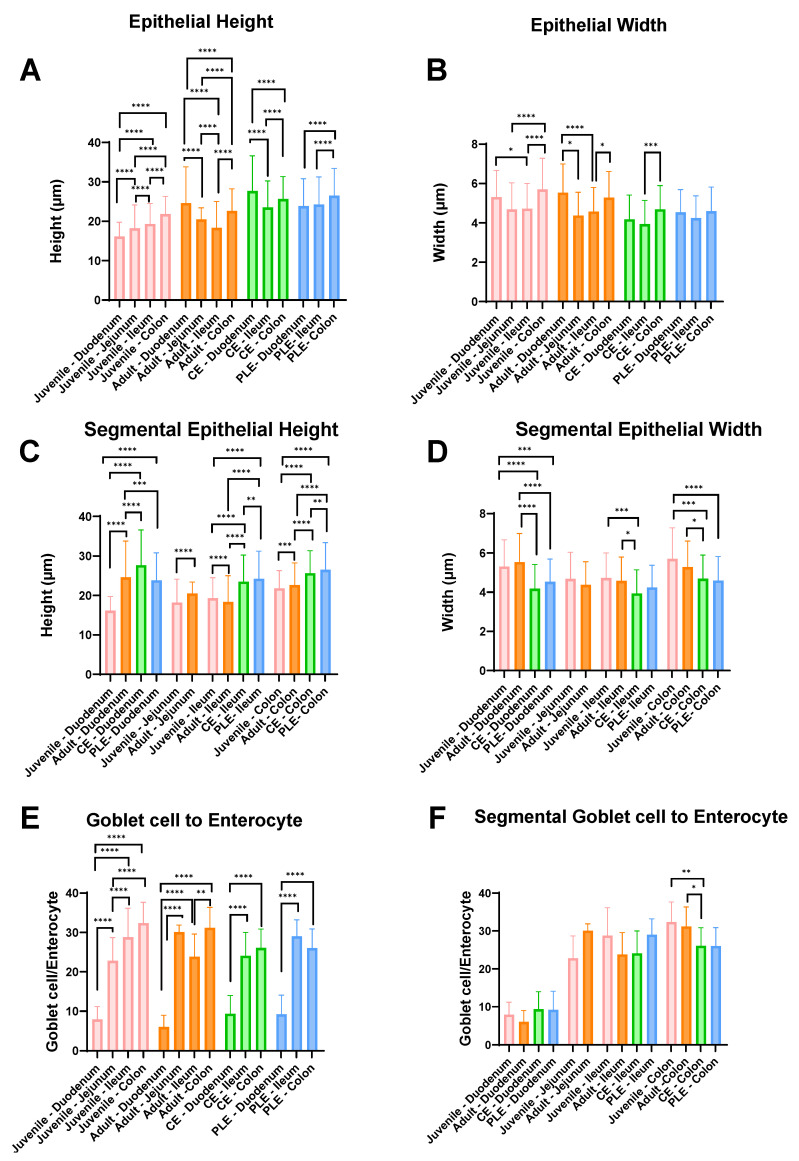
Summary results of the morphological analysis (height and width) of the enterocytes, goblet cell-to-enterocyte ratio, and the comparison by groups and intestinal segments. Healthy juvenile (pale pink), healthy adult (orange), CE (chronic enteropathy; green), PLE (protein-losing enteropathy; blue). The bars correspond to the mean, and the whiskers to the standard deviation. Significant *p*-values: <0.05 *; <0.01 **; <0.001 ***, and <0.0001 ****.

Summary results of height and width of the enterocytes and the goblet cell-to-enterocyte ratio measurements in the histopathological samples of healthy dogs (juvenile and adult) and CE and PLE dogs are summarized in Table 1 and Figure 1.

Healthy adult small intestine expressed statistically significant decreases in the small intestinal epithelial cell height measurements following the aboral direction (duodenum: 24.6 ± 9.2 µm; jejunum: 20.5 ± 2.9 µm; and ileum: 18.4 ± 6.7 µm). A similar trend was observed in the CE dogs (duodenum: 27.7 ± 8.9 µm and ileum: 23.5 ± 6.7 µm), but not in the PLE group (duodenum: 23.9 ± 7.0 µm and ileum: 24.3 ± 7.0 µm). To the contrary, healthy juvenile dogs presented with a significant increase in intestinal epithelial cell height following the aboral direction (duodenum: 16.2 ± 3.6 µm; jejunum: 18.2 ± 5.9 µm; and ileum: 19.3 ± 5.2 µm). Epithelial cell height was statistically significantly increased in the colon compared to the ileum in the healthy and the CE and PLE groups. CE dogs had increased epithelial cell height of the duodenum (27.7 ± 8.9 µm) compared to colonic epithelial cells (25.7 ± 5.7 µm). Moreover, the largest height of any epithelial cells in the large intestine was for the colonocytes in PLE dogs (26.5 ± 6.9 µm). This latter observation was further strengthened by comparing colonic epithelial cell height between all groups, with the highest enterocytes found in dogs with PLE, followed by dogs with CE and healthy dogs. The duodenum of dogs with CE showed significantly increased epithelial cell height compared to the healthy adult group, while the healthy adult group had higher duodenal epithelial cells than the PLE group.

Healthy adult dogs presented with higher enterocytes in the jejunum than juvenile dogs. The PLE group had significantly higher epithelial cells than CE dogs in the ileum, while both were taller than the ileal epithelium in the healthy dog groups. CE and PLE groups both had lower enterocyte width in the duodenal segment (CE: 4.2 ± 1.2 µm; PLE: 4.5 ± 1.2 µm) compared to the healthy adult group (5.5 ± 1.5 µm) and the healthy juvenile group (5.3 ± 1.4 µm). Following this trend, the ileal enterocytes in healthy adults (4.6 ± 1.2 µm) were wider than the ones in dogs with CE (3.9 ± 1.2 µm).

The increase in goblet cell-to-enterocyte ratio was observed in all four groups between the duodenal segment (juvenile: 7.9 ± 3.3%; adult: 6.0 ± 3.0%; CE: 9.4 ± 4.6% and PLE: 9.2 ± 4.9%) and the colonic segment (juvenile: 32.4 ± 5.3%; adult: 31.2 ± 5.2%; CE: 26.1 ± 4.8%; and PLE: 26.0 ± 4.9%). The ileum did not show any statistically significant differences in goblet cell: enterocyte ratio compared to the colon in the CE or PLE groups. Furthermore, the ileal goblet cell-to-enterocyte ratio was found to be significantly decreased (23.8 ± 5.7%) when compared to the colon in the healthy adult group (31.2 ± 5.2%).

### 3.3. Clinical Parameters

When comparing age, BW, and BCS between the CE and PLE groups, no statistical differences were found. The CCECAI index was slightly higher in PLE (11.2 ± 5.66; median: 11) than in CE dogs (9.64 ± 3.19; median: 10), but this was not statistically significant. The CCECAI of the CE and PLE dogs was correlated with variables available from the diagnostic workup of each dog as well as with the enterocyte measurements. Significant correlations are shown in Table 2.

When comparing CE and PLE groups, the PLE dogs presented with significantly lower serum cholesterol, albumin, total protein, and total calcium levels than the CE group, as expected due to the inclusion criteria for PLE dogs in this study (Table 3).

## 4. Discussion

This study assessed, for the first time, morphological features of enterocytes and the goblet cell-to-enterocyte ratio of the epithelium in the different segments of the intestine in a larger number of dogs with CE and PLE. We included 57 diseased dogs (33 CE and 24 PLE dogs) and a group of 40 healthy dogs (19 juvenile and 21 adult) as a control group. Furthermore, we compared signalment and clinical and laboratory findings in the diseased dogs and correlated the different variables with the CCECAI index.

In humans, chronic enteropathies such as CD or EED have both been shown to present with significant lymphoplasmacytic inflammatory infiltration into the lamina propria and the epithelial mucus layer, as well as shortening of the villi (so-called “flattened villi”), which is hypothesized to contribute to malabsorption and chronic wasting [19,24]. Furthermore, some recent studies performed on biopsies from people diagnosed with either CD or non-celiac gluten sensitivity clearly indicate that architectural alterations are not only found in the morphology of the villi but also in the morphology of the enterocytes themselves, with a reduced height and increased width being a hallmark of both diseases [24,25,26]. Furthermore, architectural changes of the enterocytes have also been identified in the structure of the brush border membrane, and in particular, the density of the microvilli in humans with both CD and EED [24,27].

The underlying mechanisms of these architectural changes may be related to an altered post-translational modification of the microtubules as a major element of the cytoskeleton that shifts the normal enterocyte architecture [24]. This reduction in the enterocyte mean height has also been observed when culturing jejunal biopsies from celiac patients ex vivo in the presence of gliadin and other gluten peptides [28,29]. This latter fact is intriguing, as both CD and EED are diet- and/or environmentally induced chronic enteropathies. Similarly, CE and PLE in dogs have been shown to be diet-responsive in a large percentage of cases [5,6,7,8,9,10,11,12,13,14,15,16,17,18,19,20,21,22,23,24,25,26,27,28,29,30]. It could therefore be hypothesized that the reduction in enterocyte height seen in this study in PLE dogs, is an overall adaptive response of the enterocytes in chronic lymphoplasmacytic enteropathies found across species.

Since the emergence of the WSAVA standardization for the histopathological interpretation of canine intestinal biopsies, most of the studies in canine CE have included assessments of the morphology of the intestinal villi according to the guidelines [2], but only a few have focused on the morphology of the enterocytes. One study performed on duodenal biopsies of dogs with FRE revealed higher villus stunting scores, lower villus height-to-width ratios, and ultrastructural lesions of the brush border membrane of enterocytes [31]. Furthermore, when a second endoscopy was performed in these dogs after clinical remission, resolution of the enterocyte ultrastructural lesions with an increase in microvilli density and an increase in microvillar height was observed after 6 weeks of dietary therapy [31]. For dogs in the present study, duodenal enterocytes of CE dogs had an increased height compared to the healthy adult group. However, the duodenal enterocytes of PLE dogs were significantly shorter when compared to those of healthy adult dogs. These findings could be interpreted as an initial adaptive lengthening response of the enterocytes in CE, and a shortening in the cases that are clinically more severely affected (PLE). The shortened duodenal enterocytes in the PLE dogs could well be concomitantly occurring with lesions of the brush border membrane, just like those observed previously in the study looking at FRE dogs [31]. However, further analyses using transmission electron microscopy of biopsies from PLE dogs will need to be performed to confirm this notion. However, healthy dogs are statistically younger than CE (with and without PLE) which would confound our ability to conclude if CE dogs do or do not have a higher, equivalent, or shorter enterocyte height compared to healthy adults.

Another interesting finding was that CE dogs and PLE dogs presented with decreased enterocyte width in the duodenal segment compared to healthy dogs. In addition, ileal enterocytes in the healthy adults were wider than the ones in CE dogs. These findings are opposite to those observed in the small intestinal enterocytes of human CD patients [24,25,26]. Previous studies have shown that canine CE and PLE patients have greater intestinal permeability changes than those seen in people with CD, which seem to be due to structural alterations of the tight junction (TJ) proteins in the canine disease [6,32]. It could be speculated that this loosening of interepithelial cell junctions could lead to a narrowing of the individual cell widths and contribute to the leakage of proteins into the intestinal lumen. Alterations in TJ proteins could also be affecting the large intestine, since colonic enterocytes of healthy dogs were also wider than those in CE and PLE groups in our study. In fact, when correlating the CCECAI scores with the measurements of the enterocytes, a negative correlation was found between the enterocyte mean width in the colon of the CE and PLE dogs and the CCECAI. It is interesting to note that, changes in the expression and distribution of TJ proteins have been previously described in Crohn’s disease patients and in dogs with idiopathic colitis [32,33,34] and could be speculated to result in structural changes of epithelial width. However, further studies on both small intestinal and large intestinal TJ proteins will need to be performed in order to investigate this hypothesis further.

With regards to the goblet cell-to-enterocyte ratio, the healthy juvenile dogs showed a progressive increase in the ratio in the aboral direction from the duodenum to the colon. To the authors’ knowledge, our study is the first to report enterocyte width and height as well as goblet cell numbers in juvenile healthy dogs. A similar trend was also observed in the healthy adult dogs, except for the ileal segment. The goblet cell numbers in the duodenum and colon of healthy adult dogs that we found in our study are similar to those previously described [35,36].

Alterations in the number of goblet cells have also previously been described in both CD and EED patients. CD patients present with a lower number of goblet cells overall [20,37], while EED patients present with a higher number of goblet cells when compared with healthy patients [19]. However, in our study, no differences were found in any of the small intestinal segments when counting goblet cells and enterocytes. To the contrary, colon samples of CE and PLE dogs showed a decreased goblet cell-to-enterocyte ratio compared to healthy dogs. It is important to take into consideration that the WSAVA histopathological guidelines acknowledge that measurement of goblet cells in the colonic epithelium may be artificially reduced by discharge of mucus content during the process of tissue fixation [2,9]. Thus, complementary staining techniques apart from H&E, such as AB, which we report here, could be considered to more accurately assess the number of goblet cells present in the colonic epithelium.

When comparing the CE and PLE groups, serum albumin, total protein, total calcium, and cholesterol were significantly lower in the PLE group, as expected due to the pre-determined inclusion criteria for the groups in our study. Dogs with hypoalbuminemia have previously been shown to have more severe disease and a worse prognosis among dogs with CE [1,2], which was corroborated in our study by the finding of a moderately negative correlation of serum albumin concentration with the CCECAI index. When analyzing different electrolytes, magnesium showed a moderately negative correlation with the CCECAI index. A recent study has evaluated the relationship between ionized serum magnesium and calcium in dogs with CE and PLE, concluding that hypomagnesemia might contribute to alterations in ionized calcium [38]; however, in our study, ionized magnesium and calcium concentrations were not available.

In some studies, mild non-regenerative anemia has also been found in canine chronic intestinal inflammation and has been attributed to chronic gastrointestinal bleeding, anemia of chronic disease or a combination of factors [39]. Hematocrit, hemoglobin, and red blood cell counts were negatively correlated with the CCECAI index. However, their values were not clinically significant in our study.

There are some limitations to this study. Most of the samples of the healthy adult dogs were obtained from Beagle dogs, while the diseased dogs included various different breeds. Since healthy colony Beagle dogs have previously been reported to present with various chronic intestinal abnormalities, even if they do not result in overt clinical signs [40], this caveat needs to be taken into account when interpreting our findings. Additionally, the differences in the mean age between healthy adult dogs and CE/PLE dogs could be an important factor to consider, as we also observed differences between healthy juvenile and adult dogs. Thus, the observed differences between the groups could be attributed to either age or disease status, or potentially both factors in combination. Unfortunately, due to ethical concerns, only samples from healthy dogs from colony dogs were included in our study. In addition, data regarding jejunum samples need to be interpreted cautiously, as there is minimal certainty associated with the statistical evaluation because of the very small group size in the healthy adult group. Nonetheless, our results offer new insights into the architectural changes of enterocytes and their relation to goblet cell numbers in a large cohort of dogs.

## 5. Conclusions

Our results suggest that dogs with CE and PLE present with significant alterations in enterocyte morphology, particularly in the small intestine, but also in the large intestine, and that the goblet cell-to-enterocyte ratio is reduced in the colon of CE cases compared to healthy dogs. The present study provides the first morphometric information of enterocytes in chronic lymphoplasmacytic enteritis and relates them to similar findings of such chronic enteropathies in humans. Further studies using transmission electron microscopy of the mucosa are warranted to evaluate brush border membrane and intracellular abnormalities of the enterocyte microarchitecture more in depth in dogs with CE and PLE.

## Figures and Tables

**Table 1:** Summary results of height and width of the enterocytes (length in µm ± standard deviation) and the goblet cell-to-enterocyte ratio (% ± SD) measurements in healthy dogs (juvenile and adult) and CE dogs (with and without PLE).

Groups	Healthy Dogs (Mean ± SD)				CE Dogs (Mean ± SD)			
	Juvenile		Adult		CE		PLE	
Length (µm)	Height	Width	Height	Width	Height	Width	Height	Width
Duodenum	16.2 ± 3.6	5.3 ± 1.4	24.6 ± 9.2	5.5 ± 1.5	27.7 ± 8.9	4.2 ± 1.2	23.9 ± 7.0	4.5 ± 1.2
Jejunum	18.2 ± 5.9	4.7 ± 1.4	20.5 ± 2.9	4.4 ± 1.2	NA	NA	NA	NA
Ileum	19.3 ± 5.2	4.7 ± 1.3	18.3 ± 6.7	4.6 ± 1.2	23.5 ± 6.7	3.9 ± 1.2	24.3 ± 7.0	4.2 ± 1.1
Colon	21.8 ± 4.5	5.7 ± 1.6	22.6 ± 5.6	5.3 ± 1.3	25.7 ± 5.7	4.7 ± 1.2	26.5 ± 6.9	4.6 ± 1.2
**GC to E (%)**	**Juvenile**		**Adult**		**CE**		**PLE**	
Duodenum	7.9 ± 3.3		6.0 ± 3.0		9.4 ± 4.6		9.2 ± 4.9	
Jejunum	22.8 ± 5.9		30.1 ± 1.8		NA		NA	
Ileum	28.8 ± 7.4		23.8 ± 5.7		24.1 ± 5.9		29.0 ± 4.2	
Colon	32.4 ± 5.3		31.2 ± 5.2		26.1 ± 4.8		26.0 ± 4.9	

CE: chronic enteropathy; GC to E: goblet cell-to-enterocyte ratio; NA: not apply; PLE: protein-losing enteropathy; SD: standard deviation.

**Table 2:** Significant correlations between the CCECAI index and other parameters included in the study of the CE and PLE dogs.

CCECAI vs.	r	Correlation	(+/−)	*p*-Value
Hematocrit (%)	−0.540	Moderate	Negative	0.0001
Hgb (g/dL)	−0.499	Moderate	Negative	0.0008
RBC (×10^6^/µL)	−0.462	Moderate	Negative	0.0027
Colonocyte mean width (µm)	−0.483	Moderate	Negative	0.0044
Magnesium (mg/dL)	−0.434	Moderate	Negative	0.0023
Calcium (mg/dL)	−0.410	Moderate	Negative	0.0034
Albumin (g/dL)	−0.384	Moderate	Negative	0.0077
BCS	−0.295	Low	Negative	0.0275
CIBDAI score	0.947	High	Positive	<0.0001

BCS: body condition score; CIBDAI: clinical IBD activity index; Hgb: hemoglobin; RBC: red blood cells.

**Table 3:** Comparison between CE and PLE dogs in cholesterol, albumin, total proteins, and total calcium serum levels.

Parameters	CE (Mean ± SD)	PLE (Mean ± SD)	*p*-Value	q-Value
Cholesterol (mg/dL)	197 ± 84.49	122.6 ± 49.22	0.001	0.020
Albumin (g/dL)	3.22 ± 0.52	1.47 ± 0.25	<0.001	<0.001
Total protein (g/dL)	5.99 ± 0.54	3.38 ± 0.59	<0.001	<0.001
Calcium (g/dL)	10.15 ± 0.88	7.85 ± 1.51	<0.001	<0.001

CE: chronic enteropathy; PLE: protein-losing enteropathy.

## Data Availability

The data are available in the form of supplementary material published by the DataShare service of Iowa State University (CITE).
